# Flow‐suppressed 2D spin‐echo imaging with high tolerance to B_1_ inhomogeneity using hyperbolic secant pulses

**DOI:** 10.1002/mrm.70032

**Published:** 2025-08-11

**Authors:** Jae‐Youn Keum, Jeong Hee Yoon, Michael Garwood, Jang‐Yeon Park

**Affiliations:** ^1^ Department of Intelligent Precision Healthcare Convergence Sungkyunkwan University Suwon Republic of Korea; ^2^ Department of Radiology Seoul National University Hospital and College of Medicine Seoul Republic of Korea; ^3^ Center for Magnetic Resonance Research, Department of Radiology University of Minnesota Minneapolis Minnesota USA; ^4^ Department of Biomedical Engineering Sungkyunkwan University Suwon Republic of Korea

**Keywords:** B_1_ insensitivity, DWI, flow suppression, hyperbolic secant pulse, spin‐echo MRI

## Abstract

**Purpose:**

To demonstrate flow‐suppressed two‐dimensional (2D) spin‐echo and spin‐echo diffusion echo‐planar imaging (EPI) sequences using hyperbolic secant (HS) pulses for both π/2 excitation and π refocusing.

**Theory and Methods:**

A theoretical framework to derive phase dispersion of moving spins under π/2 excitation and π refocusing using HS pulses was described. Numerical simulations were performed to verify the validity of the theoretical analysis. All experiments were performed on a 3T clinical scanner. Phantom and human‐brain imaging was performed using 2D spin‐echo sequence, and liver imaging was performed using 2D spin‐echo diffusion EPI. The signal‐to‐noise ratio and residual blood flow signal of the proposed sequences were compared with those of conventional spin‐echo sequences using sinc pulses.

**Results:**

Results from human brain and liver images demonstrated that the proposed method substantially reduced blood flow artifacts. In the brain, venous blood flow was suppressed more effectively with the proposed method than with conventional spin‐echo sequence using presaturation. In the liver, as compared with spin‐echo sequence using sinc pulses, the proposed method showed noticeable attenuation of bright blood signals at low *b*‐values, whereas the overall tissue signal in peripheral regions was higher. The signal‐to‐noise ratio was enhanced by 10% to 30%, indicating improved B_1_ tolerance due to the adiabatic π refocusing HS pulse.

**Conclusion:**

Flow suppression and partial B_1_ insensitivity were achieved by replacing sinc pulses with HS pulses in conventional 2D spin‐echo imaging and spin‐echo diffusion EPI sequences. This approach may be particularly useful in various applications requiring reduced vascular signal contamination, such as liver and brain imaging.

## 1 INTRODUCTION

In conventional two‐dimensional (2D) Cartesian spin‐echo imaging, blood flow artifacts are sometimes observed, particularly along the phase‐encoding direction.^1^, ^2^ For example, ghosting artifacts caused by blood flow can hinder lesion detection in postcontrast T_1_‐weighted brain imaging.^1^ To reduce these flow artifacts, flow compensation using gradient moment nulling or flow suppression using presaturation pulses have been used.^3^, ^4^, ^5^ Presaturation techniques are usually preferred over gradient moment nulling because they do not require modification to the main sequence. However, as preparation modules, they require additional radiofrequency (RF) pulses before the main sequence, which may result in longer scan time, higher specific absorption rate, and possibly insufficient suppression of relatively slow blood flow signals.^6^

Flow artifacts have also been reported in diffusion‐weighted imaging (DWI). DWI has been widely used in clinical applications due to its high sensitivity to tissue microstructure.^7^, ^8^, ^9^ Among its applications, liver DWI using a spin‐echo diffusion echo‐planar imaging (EPI) sequence is one of the popular clinical applications of DWI.^10^, ^11^ However, liver DWI is highly susceptible to cardiac and respiratory motions, particularly in the left lobe of the liver.^12^, ^13^, ^14^ Despite using single‐shot spin‐echo EPI, bulk motion induces spin phase dispersion, resulting in large signal loss.^14^ To mitigate motion‐induced artifacts, motion‐compensated diffusion‐weighted gradients using gradient moment nulling (e.g., bipolar diffusion‐weighted gradients) have been implemented,^15^ and some optimization techniques have recently been developed to minimize the minimum echo time (TE).^16^, ^17^ However, motion‐compensated diffusion‐weighted gradients can produce in‐phase spins in moving blood, and this gives rise to bright blood signals in liver DWI. Several methods have been proposed to eliminate these bright blood signals.^18^, ^19^ For instance, Van et al.^18^ proposed combining a monopolar diffusion‐weighted gradient in the anterior–posterior (A‐P) direction with bipolar gradients in other directions, leveraging findings that the A‐P direction is less affected by cardiac motion.^20^ Moreover, Zhang et al.^19^ proposed a motion‐robust and blood‐suppressed DWI sequence using a moderate first‐moment motion sensitivity (*M1*) value. In conventional Stejskal‐Tanner sequences for diffusion encoding, blood signals are well suppressed, preventing motion‐induced signal loss when applying a moderate *M1* value in the range of 0.1–0.27 s/mm with low (i.e., 50–100 s/mm^2^) *b*‐values.^21^ However, these techniques achieve partial blood suppression and limited motion compensation due to their reliance on moderate *M1* values.

In this study, we propose a flow‐suppressed 2D spin‐echo sequence using hyperbolic secant (HS) RF pulses for both π/2 excitation and π refocusing without any additional RF pulses or gradients. Then, we implement this idea in a 2D spin‐echo diffusion EPI sequence which has bipolar diffusion‐weighted gradients with motion compensation.^22^ This flow‐suppressed 2D spin‐echo imaging and spin‐echo diffusion EPI using HS pulses offers the additional benefit of high tolerance to B_1_ inhomogeneity due to the adiabatic property of the π refocusing HS pulse. In addition, this new method is highly insensitive to B_0_ inhomogeneity due to high RF pulse bandwidths of the frequency‐modulated π/2 and π pulses. The proposed method was analytically and numerically described, and its performance was demonstrated in phantom, human brain, and human liver imaging on a 3T clinical scanner.

## 2 THEORY

### 2.1 HS pulses

The HS pulse was first used in the field of magnetic resonance by Silver et al.^23^ more than three decades ago. Nowadays, the HS pulse is commonly used in MRI pulse sequences because it can produce a sharply demarcated response in frequency space, and when used as a π pulse, it can produce uniform spin inversion even when B_1_ is spatially nonuniform. Unlike square or sinc pulses with only amplitude modulation (AM), HS pulses as frequency‐swept pulses are usually expressed in terms of both AM and frequency modulation (FM) functions,^24^ which are given by

(1) $$\omega_{1}\left(\tau \right)=\omega_{1}^{\max}\text{sech}\left(\beta \tau \right)$$

and

(2) $$\begin{array}{c} \omega_{\mathrm{RF}}\left(\tau \right)-\omega_{c}=A\tanh\left(\beta \tau \right), \end{array}$$

where *ω*_1_(*τ*) is the time‐dependent pulse amplitude; *ω*_RF_(*τ*) is the time‐dependent pulse frequency in rad/s; *ω*_1_^max^ is the peak amplitude of the HS pulse in rad/s; *β* is a dimensionless truncation factor, commonly set to 5.3 so that sech(*β*) = 0.01, which implies that the HS pulse is truncated to 1% of the maximum amplitude at the edges; *A* and *ω*_*c*_ are the amplitude and the center frequency of the frequency‐sweep range in rad/s, respectively; *τ* is the normalized time for 0 ≤ *t* ≤ *T*_p_, which is defined as *τ* = 2 *t*/*T*_p_−1, where *T*_p_ is a pulse duration in seconds. The HS pulse can also be expressed in complex form combining AM and FM functions as follows:

(3) $$\begin{array}{c} \omega_{1}\left(\tau \right)=\omega_{1}^{\max}\text{sech}\left(\beta \tau \right)\;e^{\mathrm{i\varphi}\left(t\right)}, \end{array}$$

where $\varphi \left(t\right)=\int A\;\tan h\left(\beta \tau^{\prime }\right)d\tau^{\prime }=-\frac{A}{\beta }\;\ln\left(\text{sech}\left(\beta \tau \right)\right)$.

A single adiabatic full‐passage (AFP) pulse, including the HS pulse, has not been frequently used for π refocusing in a spin‐echo sequence despite its adiabatic property, because the isochromats, which are initially perpendicular to the effective magnetic field **ω**_**eff**_, produce nonlinear phase distribution across the slice width due to rotation about **ω**_**eff**_, resulting in signal loss in multislice 2D MRI.^25^ Although signal from moving spins may also be suppressed to some extent, the inherently substantial signal loss in stationary tissues limits its practical utility in multislice 2D MRI (see Supporting Information I).

Several methods have been proposed to compensate for the nonlinear phase distribution generated by a single AFP refocusing pulse, such as a spin‐echo sequence using two AFP π refocusing pulses^26^ or using a π/2 excitation FM pulse followed by an AFP refocusing pulse.^25^, ^27^, ^28^, ^29^ Cano et al.^28^ were the first to analyze a spin‐echo sequence using HS pulses for both π/2 excitation and π refocusing, but they assumed that the FM function of the HS pulse was approximately linear over the frequency‐sweep range. In contrast, Park et al.^29^ analyzed the spin‐echo sequence that uses HS pulses for both π/2 excitation and π refocusing without any additional assumptions on the FM function. Furthermore, they demonstrated that when HS pulses are used for both π/2 excitation and π refocusing, each HS pulse produces a nonlinear phase distribution resembling a quadratic function with opposite signs, and there are certain conditions that cause these linear phase distributions to cancel each other out. In this case, these conditions can be used for effective flow suppression, because during the TE, proton spins in the moving flow do not undergo this nonlinear phase compensation but rather undergo dephasing leading to signal loss.^30^

### 2.2 Theoretical description of flow suppression in a spin‐echo sequence using π/2 HS – π HS pulses

In the presence of slice‐selective gradients in the z‐axis, the nonlinear phase distributions across the slice width generated by the π/2 excitation HS pulse (*ϕ*_π/2,HS_) after the slice‐rephasing gradient and the π refocusing HS pulse (*ϕ*_π,HS_), respectively, can be first‐order‐approximated in a quadratic form through Taylor expansion as follows^29^:

(4) $$\begin{array}{c} \phi_{\frac{\pi }{2},\mathrm{HS}}\left(z\right)=\frac{\gamma^{2}{G_{1}}^{2}T_{p,1}}{4\beta_{1}A_{1}}z^{2}-\frac{A_{1}T_{p,1}}{2\beta_{1}}\ln\left(\text{sech}\beta_{1}\right) \end{array}$$

and

(5) $$\begin{array}{c} \phi_{\pi ,\mathrm{HS}}\left(z\right)=\frac{\gamma^{2}{G_{2}}^{2}T_{p,2}}{2\beta_{2}A_{2}}z^{2}-\frac{A_{2}T_{p,2}}{\beta_{2}}\ln\left(\text{sech}\beta_{2}\right), \end{array}$$

where *G* is the amplitude of slice‐selection gradient in *T*/*m* and *γ* is the gyromagnetic ratio in rad/s/T. Subscripts 1 and 2 denote π/2 excitation and π refocusing, respectively. By applying the selected condition^29^ (i.e., [*β*_1_ = *β*_2_ = *β*, *T*_p,1_ = *T*_p,2_ = *T*_p_, *A*_1_ = 2*A*_2_ = 2*A*, *G*_1_ = 2*G*_2_ = 2*G*] [Condition II of Park and Garwood^29^]), to Eqs. (4) and (5), the phase distributions become identical as follows:

(6) $$\begin{array}{c} \phi_{\frac{\pi }{2},\mathrm{HS}}\left(z\right)=\frac{\gamma^{2}G^{2}T_{p}}{2\beta A}z^{2}-\frac{AT_{p}}{\beta }\ln\left(\text{sech}\beta \right) \end{array}$$

and

(7) $$\begin{array}{c} \phi_{\pi ,\mathrm{HS}}\left(z\right)=\frac{\gamma^{2}G^{2}T_{p}}{2\beta A}z^{2}-\frac{AT_{p}}{\beta }\ln\left(\text{sech}\beta \right). \end{array}$$

**FIGURE 1:**
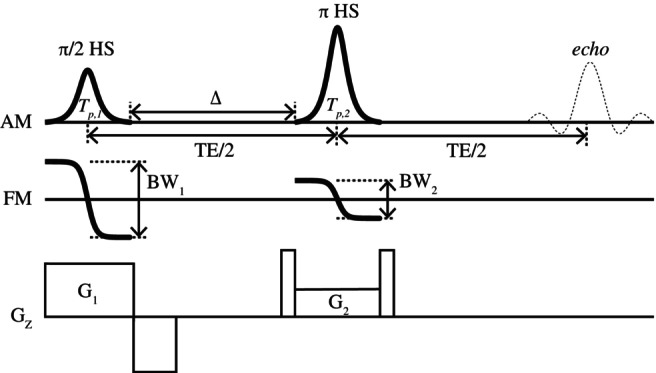
The proposed two‐dimensional spin‐echo sequence diagram using hyperbolic secant (HS) pulses for both π/2 excitation and π refocusing, satisfying Condition II to compensate for nonlinear phases across the slice (i.e., *β*_1_ = *β*_2_, *T*_p,1_ = *T*_p,2_, *BW*_1_ = 2*BW*_2_, *G*_1_ = 2*G*_2_). Only slice‐selection gradients are illustrated, along with the amplitude modulation (AM) and frequency modulation (FM) functions of the HS pulses, to highlight the applied condition (Condition II). The slice‐selection gradient for the π refocusing HS pulse is combined with crusher gradients. This sequence can provide high B_1_ insensitivity due to the adiabatic property of the π refocusing HS pulse. BW, bandwidth; TE, echo time.

Therefore, under this condition, for static spins, the nonlinear phases of *ϕ*_π/2,HS_ and *ϕ*_π,HS_ cancel each other, and a 2D spin‐echo imaging sequence satisfying this condition is illustrated in Figure 1.

In addition, to facilitate theoretical analysis, the following three assumptions were made about the moving spin of the blood flow:

- Spin isochromats are excited/refocused instantaneously at the center of the HS pulse duration;
- The quadratic phase distributions, Eqs. (6) and (7), are instantaneously formed exactly at the center of the HS pulse duration; and
- Due to the relatively short pulse duration compared with TE, the quadratically accumulated phase of the moving spins, induced by the slice‐selection gradient during HS excitation and refocusing, is canceled out by the slice‐rephasing gradient following excitation and by the slice‐selection gradient itself for refocusing, which is symmetric about the center of the refocusing HS pulse.

For moving spins, the total phase at TE *ϕ*_total_(*z*) can be expressed as the sum of distinct contributions:

(8) $$\begin{array}{cc} \;\phi_{\text{total}}\left(z\right) & =\phi_{\frac{\pi }{2},\mathrm{HS}}\left(z\right)+\phi_{G_{\mathrm{ex}}}\left(z\right)+\phi_{G_{\text{reph}}}\left(z\right)+\phi_{\Delta }\left(z\right)\\ & \;+\phi_{G_{\mathrm{re},1}}\left(z\right)+\phi_{\pi ,\mathrm{HS}}\left(z\right)+\phi_{G_{\mathrm{re},2}}\left(z\right)+\phi_{\mathrm{TE}}\left(z\right), \end{array}$$

where *ϕ*_Gex_ is an additional phase accumulated by slice‐selection gradient following excitation; *ϕ*_Greph_ is an additional phase accumulated by slice‐rephasing gradient; *ϕ*_Δ_ is the phase accumulated during the time interval Δ (see Figure 1); *ϕ*_Gre,1_ is an additional phase accumulated by slice‐selection gradient before refocusing; *ϕ*_Gre,2_ is the additional phase accumulated by slice‐selection gradient following refocusing; and *ϕ*_TE_ is the phase accumulated from the end of the π refocusing HS pulse to TE. Among these contributions, *ϕ*_Δ_ and *ϕ*_TE_ can be reasonably neglected, as there are no gradients applied along the z‐direction during the time interval Δ and the time between the end of the refocusing HS pulse and TE. In addition, under Assumption (iii), *ϕ*_Gex_ + *ϕ*_Greph_ and *ϕ*_Gre,1_ + *ϕ*_Gre,2_ are assumed to cancel out, simplifying *ϕ*_total_ (*z*) as *ϕ*_total_ (*z*) = *ϕ*_π/2_ + *ϕ*_π_. Moreover, with the Assumptions (i) and (ii), Eq. (6) is formed at *t* = 0 and Eq. (7) is formed at *t* = TE/2. Therefore, the total phase at TE for moving spins can be obtained by adding Eqs. (6) and (7) shifted by *v⋅*TE and *v⋅*TE/2 (Figure S2), respectively, which are given by

(9) $$\begin{array}{c} \phi_{\frac{\pi }{2},\mathrm{HS}}\left(z\right)=-\frac{\gamma^{2}G^{2}T_{p}}{2\beta A}{\left(z-2d\right)}^{2}+\frac{AT_{p}}{\beta }\ln\left(\text{sech}\beta \right) \end{array}$$

and

(10) $$\begin{array}{c} \phi_{\pi ,\mathrm{HS}}\left(z\right)=\frac{\gamma^{2}G^{2}T_{p}}{2\beta A}{\left(z-d\right)}^{2}-\frac{AT_{p}}{\beta }\ln\left(\text{sech}\beta \right), \end{array}$$

where *d* is the distance that the flow travels during the time between TE and the center of the π refocusing HS pulse and is given by *v⋅*TE/2. It should be noted that Eq. (9) has the opposite polarity of Eq. (6), because the phase is reversed due to the π refocusing pulse. Therefore, by adding Eqs. (9) and (10), the overall phase distribution at TE can be expressed as

(11) $$\begin{array}{c} \phi_{\text{total}}\left(z\right)=\frac{\gamma^{2}G^{2}T_{p}d}{\beta A}z-\frac{3\gamma^{2}G^{2}T_{p}d^{2}}{2\beta A}. \end{array}$$

Because the bandwidth corresponding to the slice thickness *Δz* is given by *γGΔz* and is equal to 2*A*, by replacing *γ* ^2^ *G* ^2^ = 4*A* ^2^/(*Δz*)^2^, Eq. (11) becomes

(12) $$\begin{array}{c} \phi_{\text{total}}\left(z\right)=\frac{4AT_{p}d}{\beta {\left(\Delta z\right)}^{2}}z-\frac{6AT_{p}d^{2}}{\beta {\left(\Delta z\right)}^{2}}. \end{array}$$

To evaluate the effect of the phase Eq. (12) on the magnitude of the intravoxel signal, we assume that the intravoxel magnetization is uniform (*M*) and that the intravoxel phase variation depends only on the z‐axis, in which case the magnitude of the intravoxel signal (*S*_voxel_) at TE can be expressed as

(13) $$S_{\text{voxel}}=\int_{-\frac{\Delta z}{2}+2d}^{\frac{\Delta z}{2}+d}Me^{i\phi_{\text{total}}\left(z\right)}\mathrm{dz}.$$

Here, the lower and upper bounds of this integral correspond to the lower and upper bounds of the slice width applied to the moving spins. The spins excited by the π/2 excitation HS pulse are in the range [−Δ*z*/2, Δ*z*/2], and because the spins in the flow with velocity *v* travel a distance *d* during TE/2, they are refocused in the range [−Δ*z*/2 + *d*, Δ*z*/2] by the π refocusing HS pulse. These spins then move further by a distance *d* during another TE/2 so that they are in the range [−Δ*z*/2 + 2*d*, Δ*z*/2 + *d*] at *t* = TE. Therefore, Eqs. ((11), (12), (13)) are valid when Δ*z* – *d* ≥ 0, which corresponds to a velocity condition of *v* ≤ 2Δ*z*/TE. If the flow velocity exceeds 2Δ*z*/TE, the spin‐echo sequence inherently results in signal void for flowing spins.

Upon substitution of Eqs. (12) into (13), the magnitude of the intravoxel signal becomes

(14) $$\begin{array}{c} S_{\text{voxel}}=M\left|\int_{-\frac{\Delta z}{2}+2d}^{\frac{\Delta z}{2}+d}e^{i\;\left(\;\frac{4AT_{p}d}{\beta {\left(\Delta z\right)}^{2}}\right)\;z}\mathrm{dz}\;\right|, \end{array}$$

By extracting the common factor exp[(4*iAT*_*p*_ *d*/*β*(Δ*z*)^2^)⋅(3*d*/2)] and using Euler's formula, Eq. (14) is given by

(15) $$\begin{array}{c} S_{\text{voxel}}=M\frac{\beta {\left(\Delta z\right)}^{2}}{2AT_{p}d}\left|\;\sin\left[\frac{2AT_{p}d}{\beta {\left(\Delta z\right)}^{2}}\left(\Delta z-d\right)\right]\;\right|, \end{array}$$

and Eq. (15) can be rewritten in the form of a sinc function as

(16) $$\begin{array}{c} S_{\text{voxel}}=M\left(\Delta z-d\right)\left|\;\mathrm{sinc}\left[\frac{2AT_{p}d}{\beta {\left(\Delta z\right)}^{2}}\left(\Delta z-d\right)\right]\;\right|. \end{array}$$

Then, by replacing *d* with *v⋅*TE/2, Eq. (16) is expressed in terms of *v*, which is

(17) $$\begin{array}{c} S_{\text{voxel}}=M\frac{\;\mathrm{TE}}{2}\left(\frac{2\Delta z}{\mathrm{TE}}-v\right)\left|\;\mathrm{sinc}\left[\frac{AT_{p}\;{\mathrm{TE}}^{2}v}{2\beta {\left(\Delta z\right)}^{2}}\left(\frac{2\Delta z}{\mathrm{TE}}-v\right)\right]\;\right|. \end{array}$$

In Eq. (17), if pulse parameters such as *A*, *β* and *T*_*p*_, as well as sequence parameters such as TE and *Δz*, are constant, the magnitude of the intravoxel signal depends only on the flow velocity *v*. In this case, the velocity *v* that makes the signal intensity zero can be calculated from Eq. (17). The argument of the sinc function is a negative quadratic function of *v*, and is zero when *v* is 0 or 2*Δz*/TE, so it is positive in the velocity range 0 < *v* < 2*Δz*/TE, and Eq. (17) first becomes 0 as it approaches π, that is,

(18) $$\begin{array}{c} \frac{AT_{p}{\mathrm{TE}}^{2}v}{2\beta {\left(\Delta z\right)}^{2}}\left(\frac{2\Delta z}{\mathrm{TE}}-v\right)=\pi . \end{array}$$

From Eq. (18), the smallest velocity *v*_null_ that leads to signal nulling is expressed as

(19) $$\begin{array}{c} v_{\text{null}}=\left(\frac{\Delta z}{\mathrm{TE}}\right)\cdot \left(1-\sqrt{1-\frac{2\beta \pi }{AT_{p}}}\right). \end{array}$$

Here, Eq. (19) is valid only when the term inside the square root is positive or zero. In other words, the condition such as

(20) $$\begin{array}{c} BW_{\mathrm{HS},\pi }\;T_{p}\geq 2\beta \end{array}$$

must be satisfied to calculate *v*_null_ using Eq. (19), where *BW*_HS,π_ [Hz] (= 2*A/2*π) is the bandwidth of the π refocusing HS pulse. The left‐hand side of Eq. (20) indicates the dimensionless time‐bandwidth product of the π refocusing HS pulse. Thus, when this time‐bandwidth product is smaller than 2*β*, the signal intensity will not be zero at any flow velocity below 2*Δz*/TE.

We have shown through an analytical approach that the proposed sequence offers more effective flow suppression than the conventional 2D spin‐echo sequence. Furthermore, when the sequence parameters are selected, Eq. (19) can be used to estimate the blood flow velocity at which the signal intensity within the voxel becomes null, and conversely, to select appropriate sequence parameter values that can effectively suppress the target blood flow. Interestingly, Eq. (19) shows the blood signal–nulling velocity decreases with increasing pulse duration and bandwidth, suggesting that the degree of blood flow suppression depends on the time‐bandwidth product of the HS pulse.

Unlike HS pulses, sinc pulses have a fixed frequency. Therefore, the phase does not vary over time, and no additional phase is induced by sinc pulses. Consequently, Eq. (13) can be simplified as

(21) $$\begin{array}{c} S_{\text{voxel}}=M\left(\Delta z-d\right), \end{array}$$

which indicates that the signal intensity decreases linearly with increasing velocity, as the number of spins affected by both π/2 excitation and π refocusing decreases linearly.

### 2.3 Numerical simulation

In this analysis, the phase generated by the HS pulse was first‐order‐approximated in quadratic form using Taylor expansion, and the phases involved in the proposed sequence were derived under several assumptions to gain theoretical insights into the flow suppression of that sequence. However, spin isochromats are sequentially excited/refocused due to the frequency sweep of the HS pulse, forming a quadratic‐phase distribution at the end of the HS pulse. Additionally, the phase of the moving spins, which accumulates quadratically over time due to the slice‐selection gradient (i.e., ϕ=∫γGvtdt) is not perfectly canceled out. Therefore, to validate this theoretical description based on a more precise analysis, it is necessary to calculate the exact resonance time and evaluate the accumulated quadratic‐like phase caused by the slice‐selection gradient without approximation.

The phase distribution of moving spins generated by the π/2 excitation HS pulse can be derived using the analysis from previous work.^29^ In other words, the resonance of each spin isochromat is assumed to be achieved at a unique time, *t*_Ω,1_, at which the resonance offset is equal to the value of the FM function of the π/2 excitation HS pulse; thus, *t*_Ω,1_ can be expressed as

(22) $$\begin{array}{c} t_{\Omega ,1}=\frac{T_{p,1}}{4\beta_{1}}\ln\left(\frac{A_{1}+\Omega }{A_{1}-\Omega }\right)+\frac{T_{p,1}}{2}, \end{array}$$

where *Ω* is a resonance offset frequency defined as $\Omega =\gamma G_{1}z$, and the phase generated by the π/2 excitation HS pulse including the slice‐rephasing gradient can be expressed as

(23) $$\begin{array}{cc} \phi_{\frac{\pi }{2},\mathrm{HS}}\left(z,\frac{3}{2}T_{p,1}\right) & =\phi_{\frac{\pi }{2},\mathrm{HS}}\left(z,t_{\Omega ,1}\right)+\frac{\pi }{2}\\ & \;+\int_{0}^{T_{p,1}-t_{\Omega ,1}}\gamma G_{1}\left(z+\mathrm{vt}\right)\mathrm{dt}\\ & \;-\int_{T_{p,1}-t_{\Omega ,1}}^{\frac{3T_{p,1}}{2}-t_{\Omega ,1}}\gamma G_{1}\left(z+\mathrm{vt}\right)\mathrm{dt}. \end{array}$$

On the other hand, the phase generated by the π refocusing HS pulse can be expressed as

(24) $$\begin{array}{cc} \phi_{\pi ,\mathrm{HS}}\left(z,T_{p,2}\right) & =-\int_{T_{p,1}+\Delta -t_{\Omega ,1}}^{T_{p,1}+\Delta +t_{\Omega ,2}-t_{\Omega ,1}}\gamma G_{2}\left(z+\mathrm{vt}\right)\mathrm{dt}+2\phi_{\pi ,\mathrm{HS}}\left(z,t_{\Omega ,2}\right)\\ & \;+\int_{T_{p,1}+\Delta +t_{\Omega ,2}-t_{\Omega ,1}}^{T_{p,1}+\Delta +T_{p,2}-t_{\Omega ,1}}\gamma G_{2}\left(z+\mathrm{vt}\right)\mathrm{dt}. \end{array}$$

where *Δ* is a time delay between the π/2 excitation and π refocusing HS pulses, and *t*_Ω,2_ is a unique resonance time of moving spins during π refocusing. In the same manner as in the case of π/2 excitation, the time at which the excited moving spins achieved resonance during π refocusing can be obtained by

(25) $$\begin{array}{c} \gamma G_{2}\left[z+v\left(T_{p,1}+\Delta +t_{\Omega ,2}-t_{\Omega ,1}\right)\right]=A_{2}\tanh\left[\frac{2\beta_{2}}{T_{p,2}}\left(t_{\Omega ,2}-\frac{T_{p,2}}{2}\right)\right]. \end{array}$$

Because Eq. (25) basically has the form ax+b=tanh(x) in terms of *t*_Ω,2_, which is difficult to solve using elementary functions, numerical simulation can be performed to accurately evaluate Eq. (25). However, if we assume that the frequency sweep of the π refocusing HS pulse is linear in the transition region of the hyperbolic tangent function (i.e., tanh(x)≈x), *t*_Ω,2_ can be approximated as

(26) tΩ,2≈γG2z+vTp,1+Δ−tΩ,1+A2β22A2β2Tp,2−γG2z.

Using Eqs. (26) and (25) can be solved analytically with good approximation, and the total phase distributions of moving spins generated by the π/2 excitation and π refocusing HS pulses can also be derived analytically.

Using Eqs. ((23), (24), (25))–((23), (24), (25)), we performed numerical simulations using the following steps and compared the results with the theoretical description presented in the previous section. From Eq. (22), *t*_Ω,1_ was obtained, and the phase induced by the π/2 excitation HS pulse plus the slice‐rephasing gradient was calculated by Eq. (23). Then, using *t*_Ω,1_, *t*_Ω,2_ was numerically obtained by Eq. (25), and the phase induced by the π refocusing HS pulse was calculated by Eq. (24). Finally, the final phase distribution at TE was obtained by summing Eqs. (23) and (24).

**FIGURE 2:**
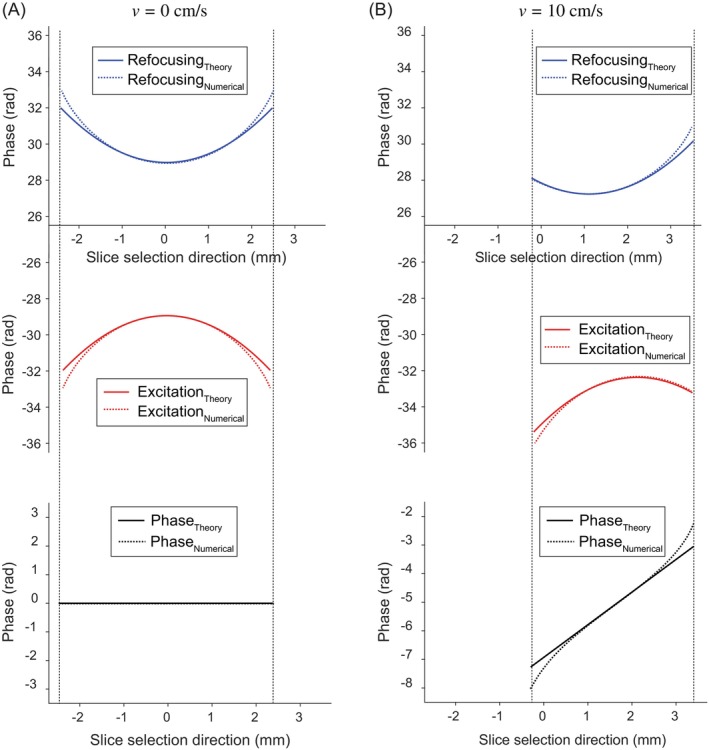
Phase distributions generated by π/2 excitation (*red*) and π refocusing (*blue*) hyperbolic secant (HS) pulses, as well as the total phase distributions at echo time (*black*), were calculated by numerical simulation and results were compared with the theoretical description for both static and moving spins. To aid understanding, Eqs. (9) and (10) was vertically shifted to match the phase range of Eqs. (23) and (24). For all cases, a slice thickness of 5 mm and echo time of 22.36 ms was used. HS pulses with *β*_1_ = *β*_2_ = 5.3, *T*_p,1_ = *T*_p,2_ = 5.12 ms, *BW*_1_/2π = 2*BW*_2_/2π = 4.14 kHz were applied for π/2 excitation and π refocusing, respectively. (A) For static spins, quadratic‐like phase distributions with opposite polarities were produced by the π/2 excitation and π refocusing HS pulses, and these phase profiles were fully compensated at echo. (B) For moving spins, a nearly linear phase distribution was formed at echo (*black dotted line*) due to the shifted quadratic‐like phase distributions with opposite polarities, which validates the theoretical description of flow suppression (*black solid line*). BW, bandwidth.

First, we obtained phase profiles for static and moving spins through numerical simulation. We computed the phase distribution at TE for 99 spins spaced 0.05 mm apart within the 5‐mm slice width in the manner described previously. As shown in Figure 2A, complete compensation of nonlinear phase profiles was achieved under the chosen condition (Condition II) for static spins. In contrast, for moving spins, a nearly linear phase distribution was formed by the sum of the shifted nonlinear phase distributions with opposite polarities (black line in Figure 2B), which validates the theoretical description for the flow suppression discussed in the previous section under the given assumptions.

**FIGURE 3:**
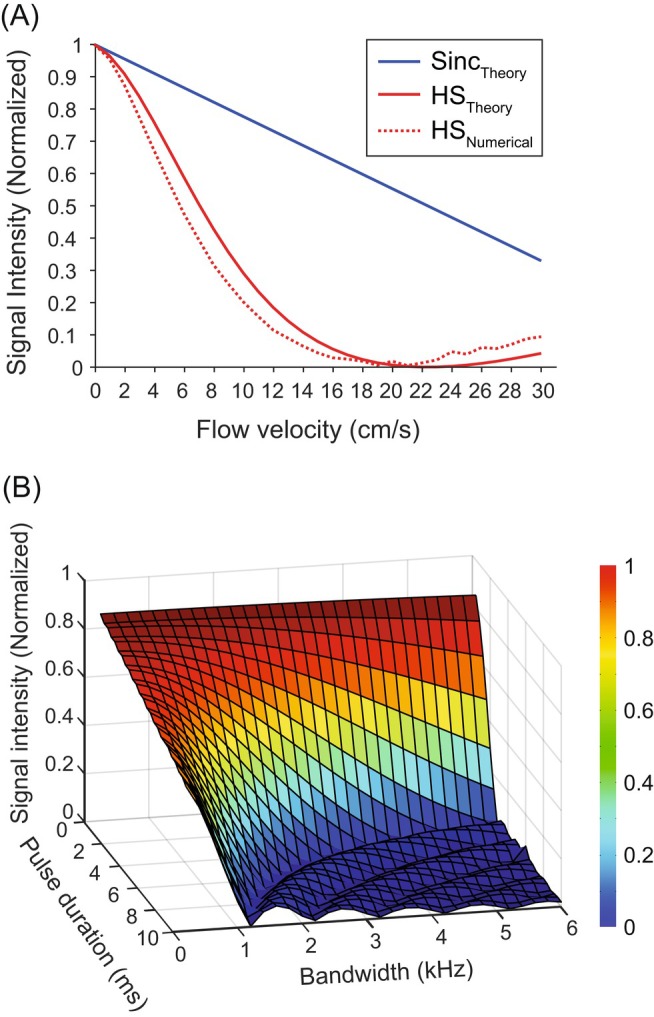
The results of the numerical simulations were illustrated. (A) Signal intensity as a function of flow velocity at echo time when sinc and hyperbolic secant (HS) pulses are used for both π/2 excitation and π refocusing. HS pulses with *β*_1_ = *β*_2_ = 5.3, *T*_p,1_ = *T*_p,2_ = 5.12 ms, and *BW*_1_/2π = 2*BW*_2_/2π = 4.14 kHz were used for π/2 excitation and π refocusing HS pulses, respectively. Scan parameters were as follows: echo time = 22.36 ms and slice thickness = 5 mm. For sinc pulses, signal intensity decreased linearly with increasing velocity. For HS pulses, signal intensity decreased more rapidly than that obtained using sinc pulses, attributed to phase dispersion caused by shifted quadratic‐like phase distributions with opposite polarities (*red dotted line*). The flow velocity that minimizes the signal intensity (i.e., 19.77 cm/s) was close to the result of the theoretical description (i.e., 22.36 cm/s). (B) Signal intensity at a flow velocity of 10 cm/s as a function of π refocusing HS pulse bandwidth and duration, when HS pulses are used for both π/2 excitation and π refocusing. Signal intensity decreased with increasing π refocusing HS pulse duration and bandwidth.

Next, we plotted the signal intensity as a function of flow velocity using the phase profiles obtained from numerical simulations and compared it with the case using sinc RF pulses. When sinc pulses were used for both π/2 excitation and π refocusing, the signal intensity decreased linearly with increasing velocity because the number of spins affected by both π/2 excitation and π refocusing decreased linearly (blue solid line in Figure 3A). This is an intrinsic effect of the conventional 2D spin‐echo sequence. However, when HS pulses were used for both π/2 excitation and π refocusing, the signal intensity decreased more rapidly than that obtained using sinc pulses (red dotted line in Figure 3A), due not only to the intrinsic effect of the 2D spin‐echo sequence, but also to the phase dispersion caused by the shifted nonlinear phase distributions (Figure 2B). Additionally, the flow velocity that minimizes the signal intensity in the numerical simulation (i.e., 19.77 cm/s) was close to the result of Eq. (19) (i.e., 22.36 cm/s), and the signal intensity behavior was consistent with that obtained through the theoretical description (red solid line in Figure 3A). We also investigated the signal intensity as a function of bandwidth (*BW*_2_) and duration (*T*_p,2_) of the π refocusing HS pulse for a given velocity (i.e., 10 cm/s). As shown in Figure 3B, a more intense flow‐suppression effect was observed as the bandwidth and duration of the π refocusing HS pulse increased. Moreover, the shape of the signal intensity as a function of pulse duration and bandwidth is similar to the shape of a sinc function, which is also consistent with Eq. (17) in the theoretical description.

These results not only validate the theoretical description presented in the previous section but also show that the theoretical description provides conceptual insights into understanding the flow suppression of the proposed spin‐echo sequence using HS pulses.

### 2.4 Application to spin‐echo diffusion EPI

So far, we have analyzed the effect of flow suppression when implementing a conventional 2D spin‐echo sequence using HS pulses for both spin excitation and refocusing. This analysis can also be applied to 2D spin‐echo EPI sequences that generate spin echoes using π/2 excitation and π refocusing HS pulses. The flow‐suppression effect observed in the 2D spin‐echo sequence using HS pulses would be particularly beneficial in DWI, especially when combined with motion‐compensated diffusion‐weighted gradients.

**FIGURE 4:**
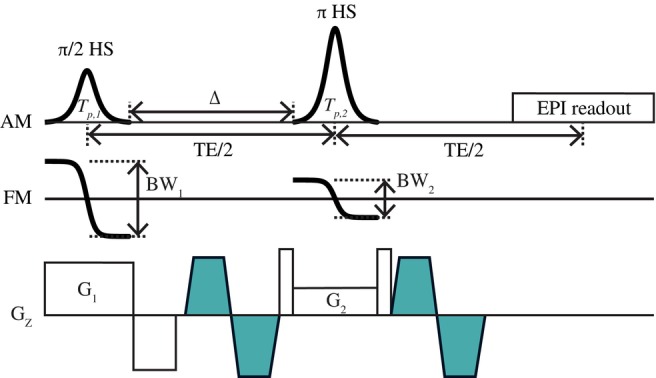
The proposed two‐dimensional spin‐echo echo‐planar imaging (EPI) sequence diagram using hyperbolic secant (HS) pulses for both π/2 excitation and π refocusing, satisfying Condition II to compensate for nonlinear phases across the slice (i.e., *β*_1_ = *β*_2_, *T*_p,1_ = *T*_p,2_, *BW*_1_ = 2*BW*_2_, *G*_1_ = 2*G*_2_). A bipolar diffusion‐weighted gradient, representing a basic form of motion‐compensated diffusion‐weighted gradients, was used for simplicity (*cyan*). Only slice‐selection‐direction diffusion‐encoding gradients are illustrated to highlight the applied condition (Condition II). The sequence was robust to B_0_ inhomogeneity effects due to relatively high radiofrequency pulse bandwidth and exhibits high B_1_ insensitivity due to the adiabatic property of π refocusing HS pulse. AM, amplitude modulation; BW, bandwidth; FM, frequency modulation; TE, echo time.

Therefore, to achieve both motion compensation and blood flow suppression in DWI, we implemented conventional bipolar diffusion‐weighted gradients in the proposed 2D spin‐echo EPI sequence using HS pulses. The proposed sequence can have the additional benefit of partial B_1_ insensitivity due to the adiabatic property of the π refocusing HS pulse. The same Condition II (i.e., [*β*_1_ = *β*_2_, *T*_p,1_ = *T*_p,2_, *A*_1_ = 2*A*_2_, *G*_1_ = 2*G*_2_]) was used for the spin‐echo EPI sequence using for HS pulse parameters as in the 2D spin‐echo sequence using HS pulses mentioned previously. The diagram of the proposed 2D spin‐echo EPI sequence was illustrated in Figure 4.

## 3 METHODS

First, spherical phantom and human brain imaging were performed using a body coil for RF transmission and BioMatrix Head/Neck 64‐channel coil for signal reception on a 3T clinical scanner (MAGNETOM Prisma; Siemens Healthineers, Erlangen, Germany) to verify the flow‐suppression effect and partial B_1_‐insensitivity of the proposed 2D spin‐echo sequence. For the π/2 excitation and π refocusing, HS pulses with *β*_1_ = *β*_2_ = 5.3, *T*_p,1_ = *T*_p,2_ = 5.12 ms, and *BW*_1_/2π = 2*BW*_2_/2π = 4.14 kHz were used, respectively. For comparison, the same experiment was also performed using 5‐lobe sinc pulses with *T*_*p*_ = 3.07 ms instead of the HS pulses. The scan parameters were as follows: For phantom imaging, repetition time (TR) = 500 ms, TE = 27 ms, and matrix size = 192 × 192; for human brain imaging, TR = 800 ms, TE = 22.36 ms, and matrix size = 256 × 256; for both imaging, field of view (FOV) = 216 × 216 mm^2^ and slice thickness = 5 mm. To compare the efficiency of venous flow suppression in human brain imaging, 2D spin‐echo imaging using sinc pulses was also performed with presaturation applied to a 5‐cm slab superior to the axial slices or anterior to the coronal slices. Eight axial slices including the superior sagittal sinus (SSS) and eight coronal slices including the SSS and internal cerebral vein (ICV) were acquired. To quantitatively assess flow suppression, SNR was measured in square regions within the SSS and ICVs.

Next, human liver imaging was performed using a body coil for RF transmission and 18‐channel receive‐only surface coil for signal reception on the same 3T scanner to demonstrate the flow‐suppression effect and partial *B*_1_‐insensitivity of the proposed 2D spin‐echo EPI sequence in DWI. For π/2 excitation and π refocusing, HS pulses with *β*_1_ = *β*_2_ = 5.3, *T*_p,1_ = *T*_p,2_ = 5.12 ms and *BW*_1_/2π = 2*BW*_2_/2π = 6.13 kHz were used, respectively. The same experiment was repeated using 5‐lobe sinc pulses with *T*_p_ = 2.05 ms. For both cases, the scan parameters were as follows: TR/TE = 1200 ms/80 ms, FOV = 280 × 280 mm^2^, matrix size = 128 × 128, slice thickness = 6 mm, *b*(average) = [50(2), 400(4), 800(8)] s/mm^2^, readout BW = 1502 Hz/px, acceleration factor = 2 (GRAPPA),^31^ 6/8 partial Fourier acquisition, and diffusion encoding direction = orthogonal. Fat suppression was performed using SPAIR (spectral attenuated inversion recovery).^32^ In human liver imaging, prospective respiratory gating was used. The apparent diffusion coefficient (ADC) map was calculated using the following definition:

(27) $$\begin{array}{c} \mathrm{ADC}=-\frac{\ln\left(S_{\text{high}}\right)-\ln\left(S_{\mathrm{low}}\right)}{b_{\text{high}}-b_{\mathrm{low}}}, \end{array}$$

where *S*_high_ and *S*_low_ are the signal magnitudes of DW images acquired with high *b*‐values (e.g., *b* = 800 s/mm^2^) and low *b*‐values (e.g., *b* = 50 s/mm^2^), respectively.

In all experiments using HS pulses, the power of the π refocusing HS pulse was set to be 3‐dB higher than the power required for an exact 180° flip angle to take advantage of the adiabatic property that provides tolerance to B_1_ inhomogeneity.

All human imaging was carried out with approval by the Institutional Review Board (SKKU 2021–06‐008) and consent.

## 4 RESULTS

**FIGURE 5:**
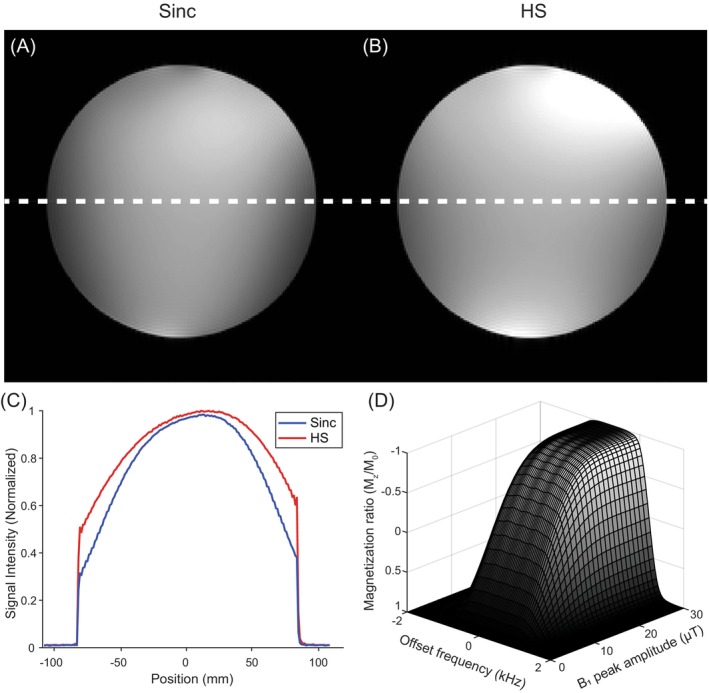
Two‐dimensional spin‐echo phantom images acquired using a head coil at 3 T. (A) The image obtained using 5‐lobe sinc pulses with *T*_*p*_ = 3.07 ms. (B) The image obtained using hyperbolic secant (HS) pulses. HS pulses with *β*_1_ = *β*_2_ = 5.3, *T*_p,1_ = *T*_p,2_ = 5.12 ms, *BW*_1_/2π = 2*BW*_2_/2π = 4.14 kHz were used for both π/2 excitation and π refocusing. To fully leverage B_1_ insensitivity, the π refocusing HS pulse was applied with nearly 3‐dB higher power than the adiabatic threshold. For both images, the scan parameters were as follows: repetition time = 500 ms, echo time = 27 ms, field of view = 216 mm × 216 mm, matrix size = 192 × 192, and slice thickness = 5 mm. (C) One‐dimensional profiles along the white line in (A) and (B). Signal‐to‐noise ratio (SNR) decreases more slowly near the periphery of the phantom image acquired with HS pulses, whereas the SNR at the center remains nearly the same in both cases, reflecting the adiabatic property of the π refocusing HS pulse. (D) Inversion profiles (*M*_*z*_/*M*_*0*_) obtained through Bloch simulation, shown as a function of offset frequency and peak radiofrequency amplitude. The same parameters as those used for π refocusing HS pulse in the phantom imaging were applied in the simulation. Spins within the pulse bandwidth experience uniform π rotation when the adiabatic condition is met.

Figure 5A,B shows phantom images obtained using 2D spin‐echo imaging with sinc pulses and HS pulses, respectively. Images acquired with HS pulses showed higher signal intensity, particularly at the periphery of the phantom, as compared to those acquired with sinc pulses. To make it clear, we compared the one‐dimensional profiles of the two images (HS vs. sinc) along the white dashed line in Figure 5A,B. As shown in Figure 5C, the signal intensities near the center were similar, but the signal intensities near the periphery of the phantom image acquired with the HS pulses were about 60% higher than those acquired with the sinc pulses, due to the adiabatic property of the π refocusing HS pulse. Figure 5D shows the inversion profiles of the longitudinal magnetization (*M*_z_) obtained from Bloch simulation as a function of frequency offset and B_1_ peak amplitude. For the simulation, the same parameters used in the phantom experiments were used for the π refocusing HS pulse. Due to the adiabatic property, spins within the pulse bandwidth undergo uniform π rotation even if the maximum amplitude of the HS pulse exceeds the minimum amplitude required for π rotation.

**FIGURE 6:**
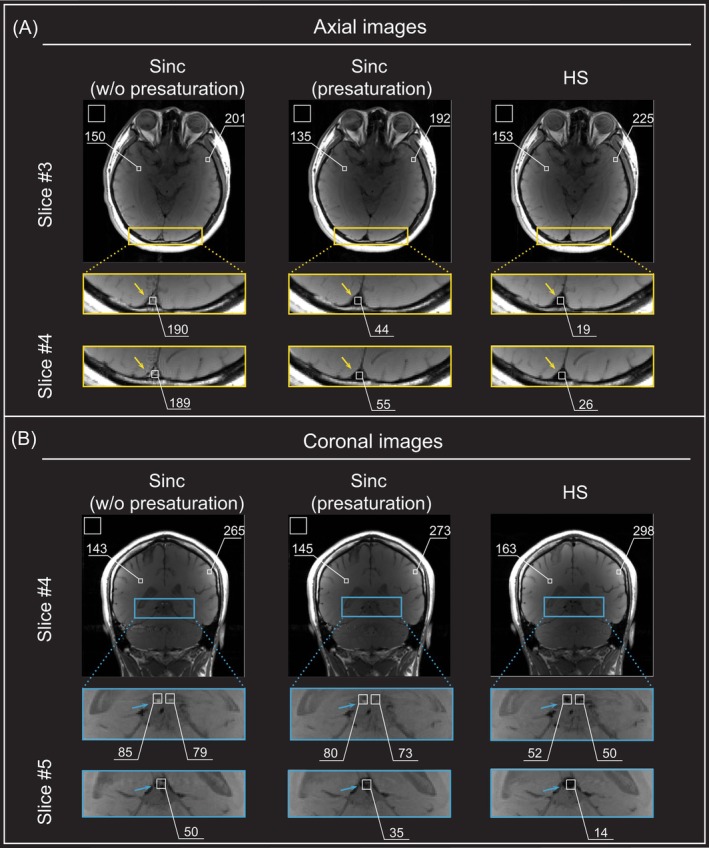
Two‐dimensional spin‐echo human brain images acquired using a head coil at 3 T. (A) Axial images obtained using 5‐lobe sinc pulses without/with presaturation and hyperbolic secant (HS) pulses without presaturation. Eight axial slices were acquired with a 20% slice gap (= 1 mm), and the third and fourth slice covering superior sagittal sinus (SSS) was chosen to compare the performance of the sequence. (B) Coronal images with the same parameters. Eight coronal slices were acquired with a 20% slice gap (= 1 mm), and the fourth and fifth slice covering internal cerebral veins (ICVs) was chosen to compare the performance of the sequence. HS pulses with *β*_1_ = *β*_2_ = 5.3, *T*_p,1_ = *T*_p,2_ = 5.12 ms, and *BW*_1_/2π = 2*BW*_2_/2π = 4.14 kHz were used for π/2 excitation and π refocusing. The numbers shown in the upper row of (A) and (B) represent the signal‐to‐noise ratio (SNR) in the white matter and gray matter (*white boxes in the brain*). SNRs were also measured within the SSS and ICVs to compare the efficiency of venous blood flow suppression. Background noise was calculated from the white box positioned in the upper left corner of the image. The image acquired with HS pulses demonstrates higher SNR, attributed to the adiabatic property of the π refocusing HS pulse. In the image acquired with sinc pulses, flow artifacts were prominent, particularly in the superior sagittal sinus, whereas signals from moving blood were effectively suppressed in the image acquired with HS pulses (*yellow arrow*).

Next, human brain images acquired using the 2D spin‐echo sequence with sinc pulses (without/with presaturation) and HS pulses (without presaturation), respectively, are shown in Figure 6. Consistent with the phantom experiments, human brain images acquired with HS pulses demonstrated higher signal intensity compared with those obtained with sinc pulses, exhibiting about 10%–20% higher SNR (white square boxes in the upper rows of Figure 6A,B). Although presaturation effectively suppressed venous blood flow in the 2D spin‐echo sequence using sinc pulses, HS pulses suppressed moving blood signals to a much greater extent and further reduced flow artifacts along the phase‐encoding direction, particularly in the SSS and ICVs, where flow velocities ranged from 5 to 20 cm/s (yellow and blue arrows in Figure 6A,B, respectively),^33^, ^34^ and also suppressed arterial flows in the cavernous internal carotid arteries (see Supporting Information III).

**FIGURE 7:**
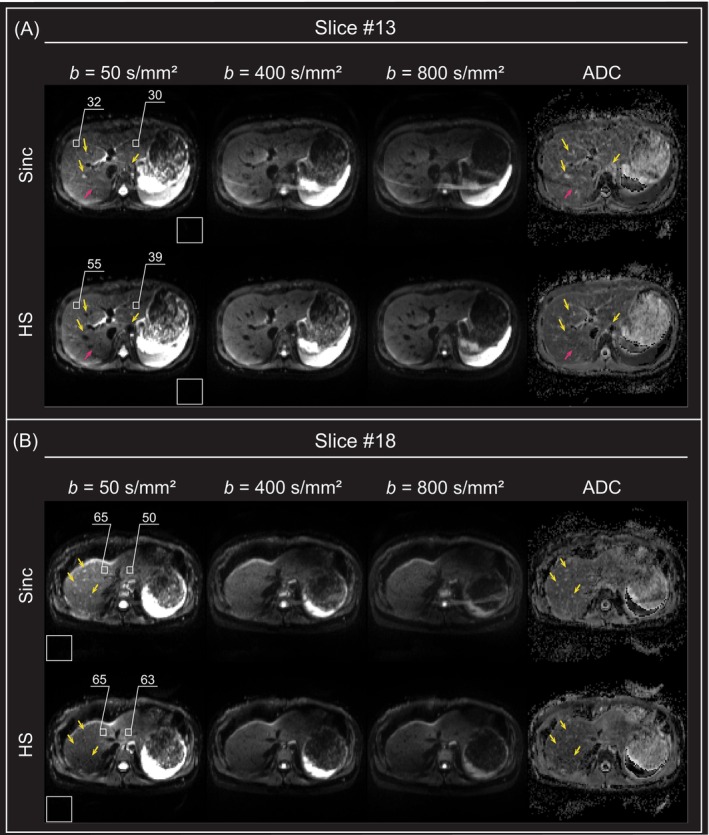
Two‐dimensional spin‐echo diffusion echo‐planar imaging (EPI) human liver images acquired using a body coil at 3 T. A bipolar motion‐compensated diffusion‐weighted gradient was used for diffusion encoding. (A) *b* = 50, 400, and 800 s/mm^2^ axial diffusion‐weighted (DW) images and apparent diffusion coefficient (ADC) maps obtained using 5‐lobe sinc pulses and hyperbolic secant (HS) pulses. (B) Axial DW images and ADC map of different slice. HS pulses with *β*_1_ = *β*_2_ = 5.3, *T*_p,1_ = *T*_p,2_ = 5.12 ms, and *BW*_1_/2π = 2*BW*_2_/2π = 6.13 kHz were used for π/2 excitation and π refocusing. The numbers shown in (A) and (B) represent the signal‐to‐noise ratio (SNR) in the right and left lobes of the liver (*white boxes in the liver*). Background noise was calculated from the white box positioned in the lower left or right corner of the image. Owing to the adiabatic π refocusing HS pulse, the image acquired with HS pulses shows high SNR in the peripheral area of the liver, with better definition of abdominal structures at the periphery. Additionally, chemical shift artifacts, which were prominent along the phase‐encoding direction in (A) (*magenta arrow*), were suppressed well with HS pulses, attributed to the fat suppression effect of Condition II. In the image acquired with sinc pulses, bright blood signals were prominent, particularly in the abdominal aorta and veins, including the branches of the right portal vein and the right hepatic vein. In contrast, these signals were effectively suppressed in the image acquired with HS pulses (*yellow arrows*).

Human liver images acquired with the 2D spin‐echo EPI sequence for DWI using HS pulses were also shown in comparison to those obtained with the same sequence using sinc pulses (Figure 7). Similar to brain imaging, liver images with *b* = 50 s/mm^2^ and ADC maps acquired using sinc pulses displayed bright signals from moving blood flow, whereas those acquired with HS pulses showed effective suppression of these signals, particularly in the abdominal aorta and veins, including the branches of the right portal vein and the right hepatic vein (yellow arrows in Figure 7A,B). Consistent with Figure 6, liver images acquired with HS pulses suppressed moving blood signals to a much greater extent than those acquired with sinc pulses using presaturation (see Supporting Information IV). The flow suppression effect, which was evident in low *b*‐value images (e.g., *b* = 50 s/mm^2^ images), gradually decreased as the *b*‐value increased (e.g., *b* = 400 and 800 s/mm^2^ images). Additionally, liver images acquired with HS pulses exhibited approximately 20–30% higher SNR in the peripheral abdominal region including the left lobe of the liver than images acquired with sinc pulses, allowing clearer visualization of peripheral structures, especially in Segment III, IVb, and V in liver anatomy (first column in Figure 7A,B). Moreover, the chemical shift artifacts that were prominent along the phase‐encoding direction (A‐P) when using the sinc pulses were diminished when using the HS pulses due to the fat suppression effect of Condition II (magenta arrow in Figure 7A).

## 5 DISCUSSION

In this work, we proposed a flow‐suppressed 2D spin‐echo sequence and a spin‐echo EPI sequence using HS pulses for both π/2 excitation and π refocusing. The proposed sequences were not only effective in suppressing blood flow but also could provide a degree of insensitivity to B_1_ inhomogeneity by using π refocusing HS pulses with adiabatic property. Although only HS pulses were used in this study, in principle, flow suppression can be achieved with any frequency‐selective RF pulse capable of generating a quadratic‐like phase distribution. Additionally, in diffusion‐weighted spin‐echo EPI using HS pulses for both π/2 excitation and π refocusing, flow suppression can be achieved even in the presence of bipolar diffusion‐weighted gradients, as well as other motion‐compensated diffusion‐weighted gradients based on gradient moment nulling, such as CODE (convex optimized diffusion encoding) gradients^16^ and ODGD (optimized diffusion‐weighting gradients).^17^

An additional advantage of this type of flow suppression is that parameters can be optimized to selectively suppress specific blood flow velocities. Furthermore, when targeting slow blood flow (e.g., about 2.5 cm/s), the proposed sequences can be more robust to B_0_ inhomogeneity due to the relatively high RF pulse bandwidth (e.g., *BW*_1_/2π = 2*BW*_2_/2π = 6.13 kHz). In addition, from a practical viewpoint, the proposed sequences offer the benefit of requiring no additional RF pulses or gradients, except replacing the conventional RF pulses with HS pulses.

According to the analysis of Park et al.,^29^ certain conditions can be derived under which the nonlinear phase distributions of *ϕ*_π/2,HS_ and *ϕ*_π,HS_ cancel each other, such as [*β*_1_ = *β*_2_, *T*_p,1_ = 2*T*_p,2_, *A*_1_ = *A*_2_, *G*_1_ = *G*_2_] (Condition I), [*β*_1_ = *β*_2_, *T*_p,1_ = *T*_p,2_, *A*_1_ = 2*A*_2_, *G*_1_ = 2*G*_2_] (Condition II), and [*β*_1_ = 0.5*β*_2_, *T*_p,1_ = *T*_p,2_, *A*_1_ = *A*_2_, *G*_1_ = *G*_2_] (Condition III). Among these conditions, Condition II (*β*_1_ = *β*_2_, *T*_p,1_ = *T*_p,2_, *A*_1_ = 2*A*_2_, *G*_1_ = 2*G*_2_) was chosen in this study for several reasons. First, Condition III was excluded because of higher truncation factor 2*β*, which creates a wide transverse magnetization transition region and requires higher refocusing pulse power. Next, when comparing Conditions I and II, Condition II can achieve a shorter minimum TE than Condition I, because the duration of the excitation HS pulse is the same as the refocusing HS pulse, and thus also a shorter scan time. Finally, an additional benefit of Condition II is its fat suppressing property.^35^ In the presence of some frequency offset (*δ*) due to field inhomogeneity or chemical shift, the nonlinear phase distributions generated by the π/2 excitation and π refocusing HS pulses under Condition II cannot be fully compensated across the slice, resulting in increasing signal loss as *δ*/*BW* increases. This may be advantageous for spin‐echo EPI sequences, as chemical shift artifacts (e.g., spatial misregistration of water and fat) can often occur in EPI along the phase‐encoding direction due to the small bandwidth per pixel.

A limitation of the proposed method at this stage is that flow suppression is achieved only along the slice‐selection direction. Therefore, the slice‐selection axis should align with the dominant blood flow direction to achieve the desired degree of blood flow signal suppression. For oblique slice imaging, where slice‐selection gradients are applied along three orthogonal directions, flow suppression can be extended to all three orthogonal directions.

Although the proposed technique is effective in many cases, it may be partially susceptible to motions in some cases. The underlying mechanism affects not only the moving spins of blood flow, but also the spins influenced by bulk motions, such as respiratory and cardiac motions. Due to these issues, the proposed technique can effectively suppress bright blood signals in liver DWI, but the image quality may be somewhat degraded in terms of SNR, which may be exacerbated in the uppermost part of the liver, close to the apex of the heart and lungs, as shown in Figure 7B (see Supporting Information V). In this regard, pulse parameters such as bandwidth and pulse duration should be carefully optimized to balance blood flow suppression and motion‐susceptibility.

An interesting direction for future work on this flow‐suppressed spin‐echo imaging approach using HS pulses is its application to multiband (MB) brain imaging. MB imaging technique is widely used in brain diffusion imaging because of its ability to reduce acquisition time.^36^, ^37^, ^38^ Incorporating the MB pulse design into the proposed HS pulse‐based sequence may benefit from the partial B_1_‐insensitivity of the adiabatic π HS refocusing pulse.^39^, ^40^ The high bandwidth may also help to mitigate off‐resonance artifacts across simultaneously excited multiple slices. In addition, flow suppression can reduce blood flow artifacts within the entire 3D volume if each simultaneously excited slice is oriented to align with blood vessels. However, implementing MB HS pulses requires careful pulse design and B_1_ power management to remain within specific absorption rate limits.

When applying the proposed 2D spin‐echo EPI sequence with motion‐compensated diffusion‐weighted gradients to DWI, distinct flow‐suppression effects occur with low *b*‐values (e.g., *b* < 100 s/mm^2^), whereas the flow‐suppression effect is less pronounced at high *b*‐values (e.g., *b* > 400 s/mm^2^). This reduced flow‐suppression effect at high *b*‐values can be attributed to several factors. As the *b*‐value increases, the signal intensity of moving blood decreases more rapidly than that of surrounding tissues, because blood has a higher ADC than tissues, particularly with motion‐compensated diffusion‐weighted gradients.^41^, ^42^ Additionally, long TE associated with high *b*‐values may result in flow signal voids.

Although diffusion‐weighted EPI images are crucial for detecting liver malignancies, they are often inadequate for accurately characterizing lesions such as hepatic hemangiomas due to the T_2_ shine‐through effect when relying solely on visual assessment.^43^, ^44^ To address this issue, ADC maps have been widely used to improve lesion detection.^45^, ^46^ Low *b*‐value images (e.g., *b* = 50 s/mm^2^) are sometimes preferred, as lesions may be overlooked on images acquired with *b* = 0 s/mm^2^ due to high signal interference from adjacent intrahepatic vessels and perfusion effects.^43^, ^44^, ^47^ However, motion‐compensated gradients that cause bright blood signals in low *b*‐value images can potentially overestimate ADC and degrade ADC maps, confounding the detection and assessment of true lesions.^16^, ^17^, ^18^, ^19^ Consequently, the proposed 2D spin‐echo EPI is expected to improve diagnostic accuracy by enhancing flow suppression in low *b*‐value images obtained with motion‐compensated diffusion‐weighted gradients.

## 6 CONCLUSIONS

In summary, we proposed highly B_1_‐insensitive flow‐suppressed 2D spin‐echo imaging and spin‐echo EPI using HS pulses and demonstrated their utility through theory, simulations, and imaging of human brain and liver. Although we used conventional 2D spin‐echo sequence and spin‐echo diffusion EPI sequence types here, the proposed method can be applied to any type of spin‐echo sequence, including turbo spin‐echo sequences.

## Supporting information


**Figure S1.** Signal intensity as a function of flow velocity at echo time (TE) when using sinc (*blue line*) and hyperbolic secant (HS) (*red solid line*) pulses for both π/2 excitation and π refocusing, and when using a single HS pulse (*red dotted line*) for π refocusing. The parameters are the same as in Figure 3A.
**Figure S2.** Phase distributions generated by π/2 excitation (*red*) and π refocusing (*blue*) hyperbolic secant (HS) pulses, as well as the total phase distributions (*black*). Under the assumptions, a nearly linear phase distribution was formed at echo time (TE) due to the shifted quadratic‐like phase distributions with opposite polarities.
**Figure S3.** Phase distributions generated by π/2 excitation (*red*) and π refocusing (*blue*) hyperbolic secant (HS) pulses, as well as the total phase distributions at echo time (TE; *black*) were calculated by numerical simulation. (A) For static spins, quadratic‐like phase distributions with opposite polarities were produced by the π/2 excitation and π refocusing HS pulses, and these phase profiles were fully compensated at TE. (B) For moving spins, a nearly linear phase distribution was formed at TE (*black*) due to the shifted quadratic‐like phase distributions with opposite polarities.
**Figure S4.** Two‐dimensional spin‐echo human brain images acquired using a head coil at 3 T. Axial images obtained using 5‐lobe sinc pulses without/with presaturation and hyperbolic secant (HS) pulses without presaturation. Arterial blood signals in the cavernous internal carotid arteries were effectively suppressed in the image acquired with HS pulses (*yellow arrows*).
**Figure S5.** Two‐dimensional spin‐echo diffusion echo‐planar imaging (EPI) human liver images acquired using a body coil at 3 T. A bipolar motion‐compensated diffusion‐weighted gradient was used for diffusion encoding. Axial images obtained using 5‐lobe sinc pulses without/with presaturation and hyperbolic secant (HS) pulses without presaturation. Owing to the adiabatic π refocusing HS pulse, the image acquired with HS pulses shows high signal‐to‐noise ratio (SNR) in the peripheral area of the liver, with better definition of abdominal structures at the periphery. In the image acquired with sinc pulses, bright blood signals were prominent, particularly in the abdominal aorta and veins. In contrast, these signals were effectively suppressed in the image acquired with HS pulses (*yellow arrows*).
**Figure S6.** Signal intensity as a function of flow velocity at echo time (TE) when sinc and hyperbolic secant (HS) pulses are used for both π/2 excitation and π refocusing. HS pulses with *β*_1_ = *β*_2_ = 5.3, *T*_p,1_ = *T*_p,2_ = 5.12 ms, and *BW*_1_/2π = 2*BW*_2_/2π = 6.13 kHz were used for π/2 excitation and π refocusing HS pulses, respectively. Scan parameters were as follows: TE = 80 ms and slice thickness = 6 mm.
**Figure S7.** Signal‐to‐noise ratio (SNR) comparison of liver images acquired by two‐dimensional spin‐echo diffusion‐weighted imaging (DWI) using sinc pulses and hyperbolic secant (HS) pulses. (A) Selected square regions of interest (ROIs) in the posterior part of the right lobe of the liver. (B) Signal‐to‐noise ratio (SNR) in each slice when using sinc pulses and HS pulses. SNR was simply calculated as the average signal intensity divided by the standard deviation within the ROI.

## Data Availability

The data that support the findings of this study are available from the corresponding author upon reasonable request.
