# Supplementary material for: Flow‐suppressed 2D spin‐echo imaging with high tolerance to B1 inhomogeneity using hyperbolic secant pulses
Source: Magn Reson Med. 2025 Aug 11;95(1):172–87. doi: 10.1002/mrm.70032 (PMC12620147; doi:10.1002/mrm.70032)
Supplement: Supplementary file 1 — Figure S1. Signal intensity as a function of flow velocity at echo time (TE) when using sinc (blue line) and hyperbolic secant (HS) (red solid line) pulses for both π/2 excitation and π refocusing, and when using a single HS pulse (red dotted line) for π refocusing. The parameters are the same as in Figure 3A. Figure S2. Phase distributions generated by π/2 excitation (red) and π refocusing (blue) hyperbolic secant (HS) pulses, as well as the total phase distributions (black). Under the assumptions, a nearly linear phase distribution was formed at echo time (TE) due to the shifted quadratic‐like phase distributions with opposite polarities. Figure S3. Phase distributions generated by π/2 excitation (red) and π refocusing (blue) hyperbolic secant (HS) pulses, as well as the total phase distributions at echo time (TE; black) were calculated by numerical simulation. (A) For static spins, quadratic‐like phase distributions with opposite polarities were produced by the π/2 excitation and π refocusing HS pulses, and these phase profiles were fully compensated at TE. (B) For moving spins, a nearly linear phase distribution was formed at TE (black) due to the shifted quadratic‐like phase distributions with opposite polarities. Figure S4. Two‐dimensional spin‐echo human brain images acquired using a head coil at 3 T. Axial images obtained using 5‐lobe sinc pulses without/with presaturation and hyperbolic secant (HS) pulses without presaturation. Arterial blood signals in the cavernous internal carotid arteries were effectively suppressed in the image acquired with HS pulses (yellow arrows). Figure S5. Two‐dimensional spin‐echo diffusion echo‐planar imaging (EPI) human liver images acquired using a body coil at 3 T. A bipolar motion‐compensated diffusion‐weighted gradient was used for diffusion encoding. Axial images obtained using 5‐lobe sinc pulses without/with presaturation and hyperbolic secant (HS) pulses without presaturation. Owing to the adiabatic π refocusing HS puls [file MRM-95-172-s001.docx]

Supporting Information for

**Flow-Suppressed 2D Spin-Echo Imaging with High Tolerance to B_1_ Inhomogeneity using Hyperbolic Secant Pulses**

Jae-Youn Keum^1^, Jeong Hee Yoon^2^, Michael Garwood^3^, and Jang-Yeon Park^1,4*^

^1^Department of Intelligent Precision Healthcare Convergence, Sungkyunkwan University, Suwon, Republic of Korea.

^2^Department of Radiology, Seoul National University Hospital and College of Medicine, Seoul, Republic of Korea.

^3^Center for Magnetic Resonance Research, Department of Radiology, University of Minnesota, Minneapolis, Minnesota, U.S.

^4^Department of Biomedical Engineering, Sungkyunkwan University, Suwon, Republic of Korea.

**Supporting Information**

**Supporting Information I. Flow suppression when using a single AFP pulse in a spin-echo sequence**

A spin-echo sequence using single AFP pulse for π refocusing may be attributed to some degree of flow suppression. However, the efficiency of flow suppression is significantly lower than that of a spin-echo sequence that uses HS pulses for both π/2 excitation and π refocusing. A spin-echo sequence that uses single HS pulse for π refocusing produces a quadratic-like phase distribution across the slice for both stationary and moving spins. This is the underlying mechanism of signal loss that occurs when using a single HS pulse for refocusing.

As shown in Figure S1, a spin-echo sequence using a single HS pulse for π refocusing (red dotted line) showed some degree of flow suppression, but for stationary spins, the signal was reduced by almost half. For this reason, a single AFP pulse has not been frequently used for refocusing in spin-echo sequences and it is important to use AFP pulses, such as HS pulses, for both excitation and refocusing, which can compensate for each other’s nonlinear phase, to avoid signal loss.

**
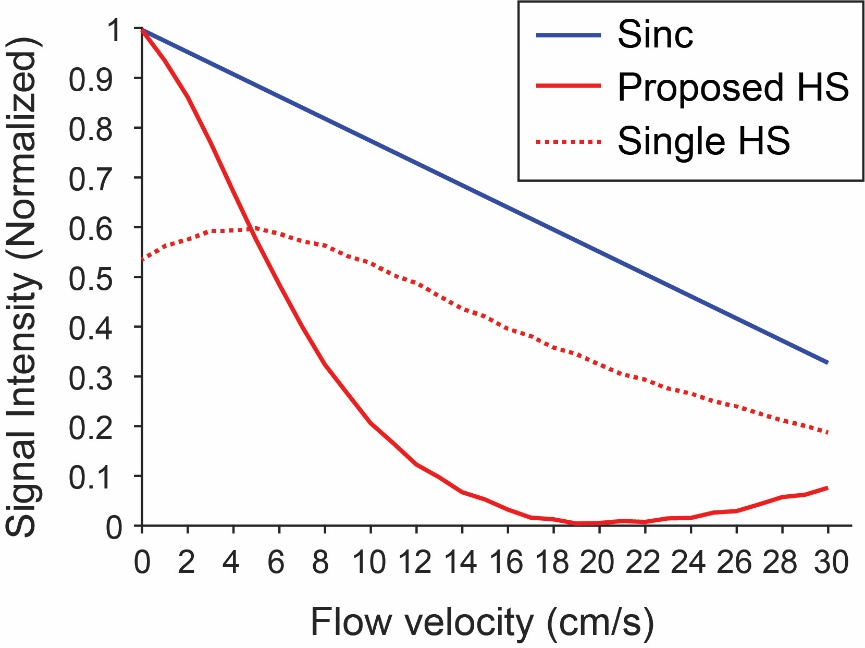
**

**Figure S1.** Signal intensity as a function of flow velocity at TE when using sinc (blue line) and HS (red solid line) pulses for both π/2 excitation and π refocusing, and when using a single HS pulse (red dotted line) for π refocusing. The parameters are the same as in Figure 3A.

**Supporting Information II. Behavior of flowing spins**


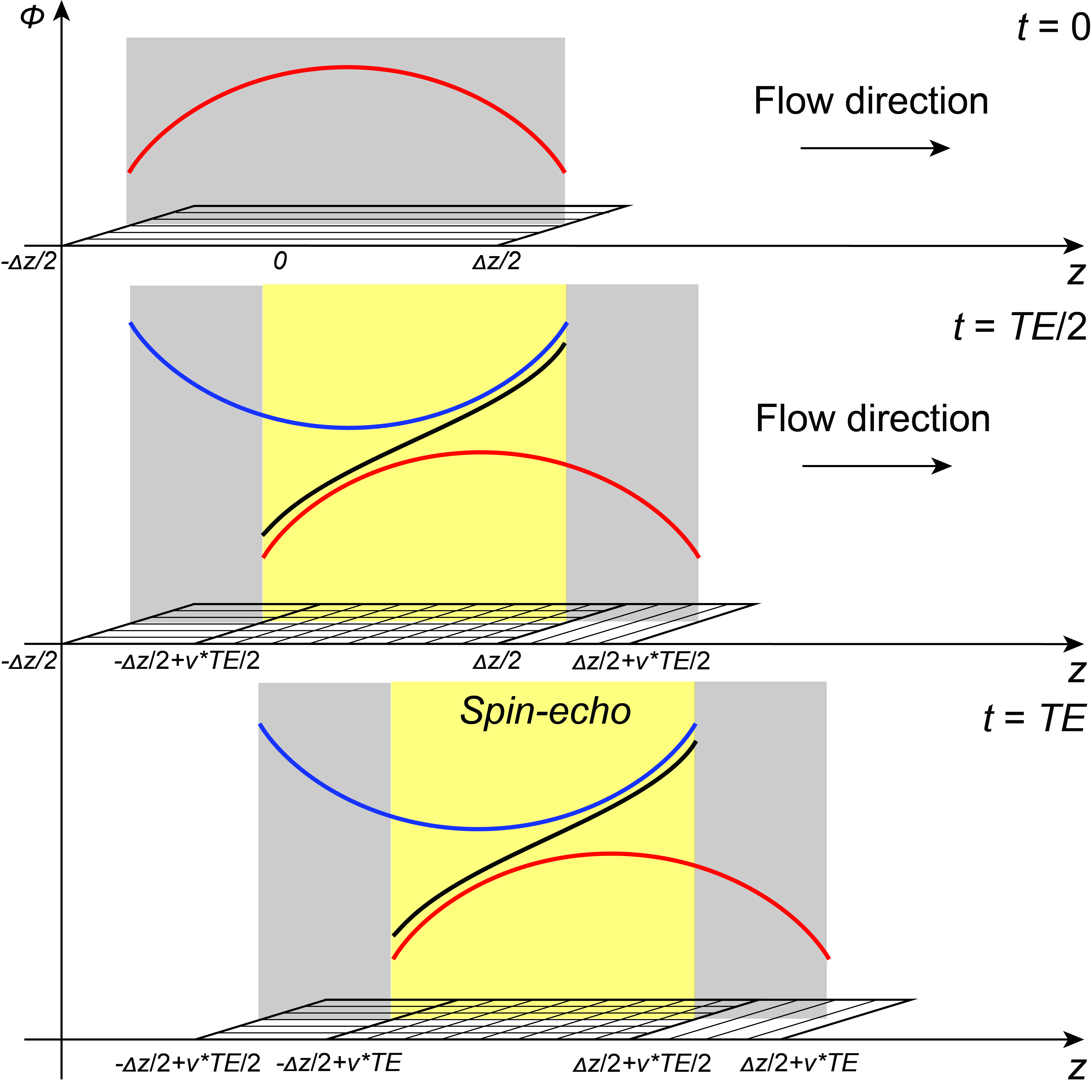


**Figure S2.** Phase distributions generated by π/2 excitation (red) and π refocusing (blue) HS pulses, as well as the total phase distributions (black). Under the assumptions, a nearly linear phase distribution was formed at echo due to the shifted quadratic-like phase distributions with opposite polarities.

**Supporting Information III. Arterial blood flow suppression**

From a clinical viewpoint, blood flow artifacts caused by arterial pulsations may be more detrimental to accurate disease diagnosis than blood flow artifacts due to venous flow (Song JW. Flow-Related Artifacts and Pitfalls in Magnetic Resonance Imaging/Angiography in Neuroradiology. Annual Meeting ISMRM 2016, Singapore). Fortunately, the proposed technique suppresses all blood flow signals passing through the slice and can also be used to suppress arterial blood flow artifacts. As shown in Figure S3B below, since the directions of venous and arterial blood flow are opposite, the quadratic-like phase distribution shifts in the opposite direction, forming a linear distribution with opposite polarity in TE (see Figure 2B).

The arterial blood flow suppression by the proposed technique is shown in Figure S4. HS pulses suppressed arterial blood signals to a much greater extent, particularly in the cavernous internal carotid arteries (cICA, yellow arrows in Figure S4).

**
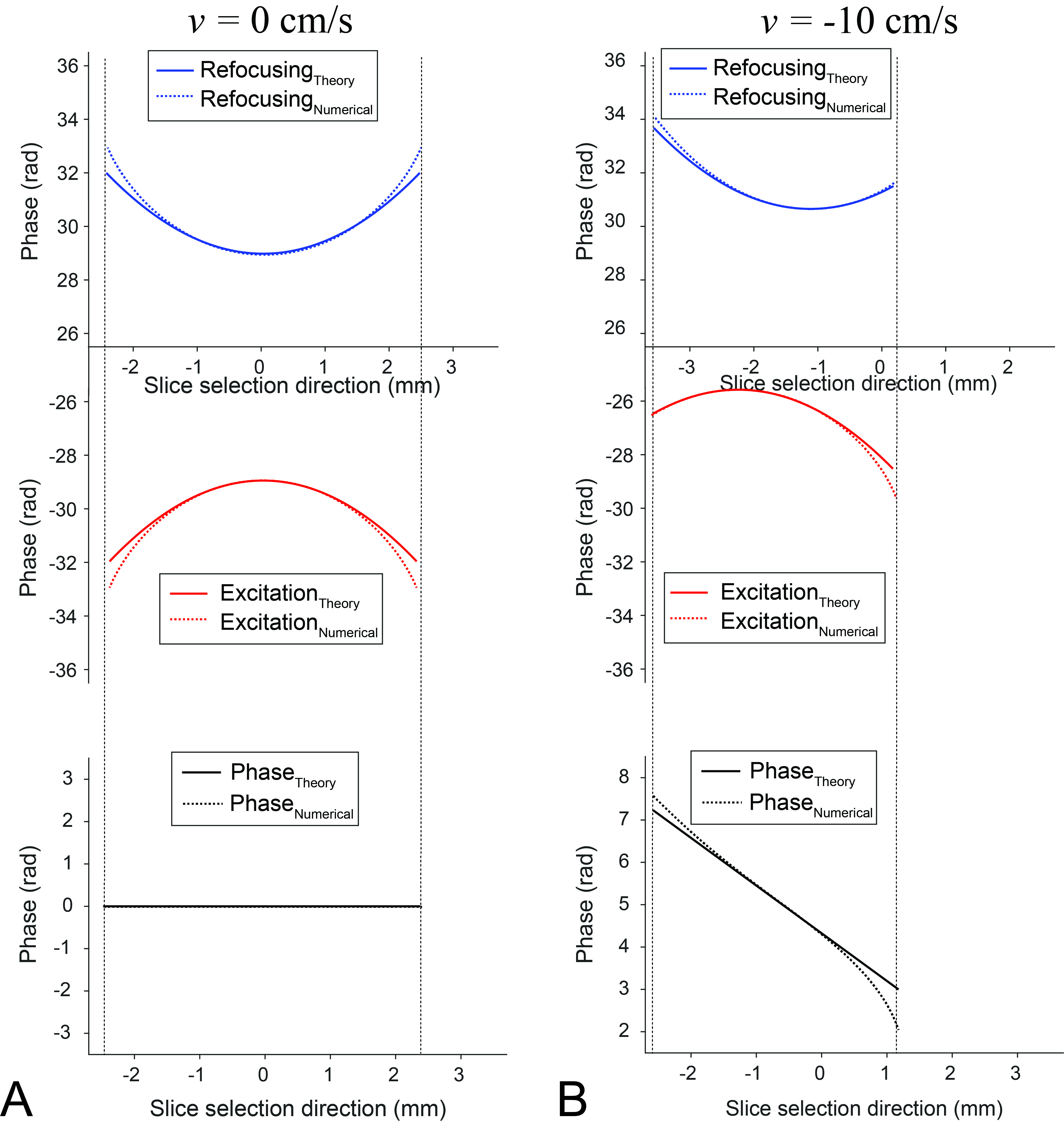
**

**Figure S3.** Phase distributions generated by π/2 excitation (red) and π refocusing (blue) HS pulses, as well as the total phase distributions at TE (black), were calculated by numerical simulation. (A) For static spins, quadratic-like phase distributions with opposite polarities were produced by the π/2 excitation and π refocusing HS pulses, and these phase profiles were fully compensated at TE. (B) On the other hand, for moving spins, a nearly linear phase distribution was formed at TE (black) due to the shifted quadratic-like phase distributions with opposite polarities.

**
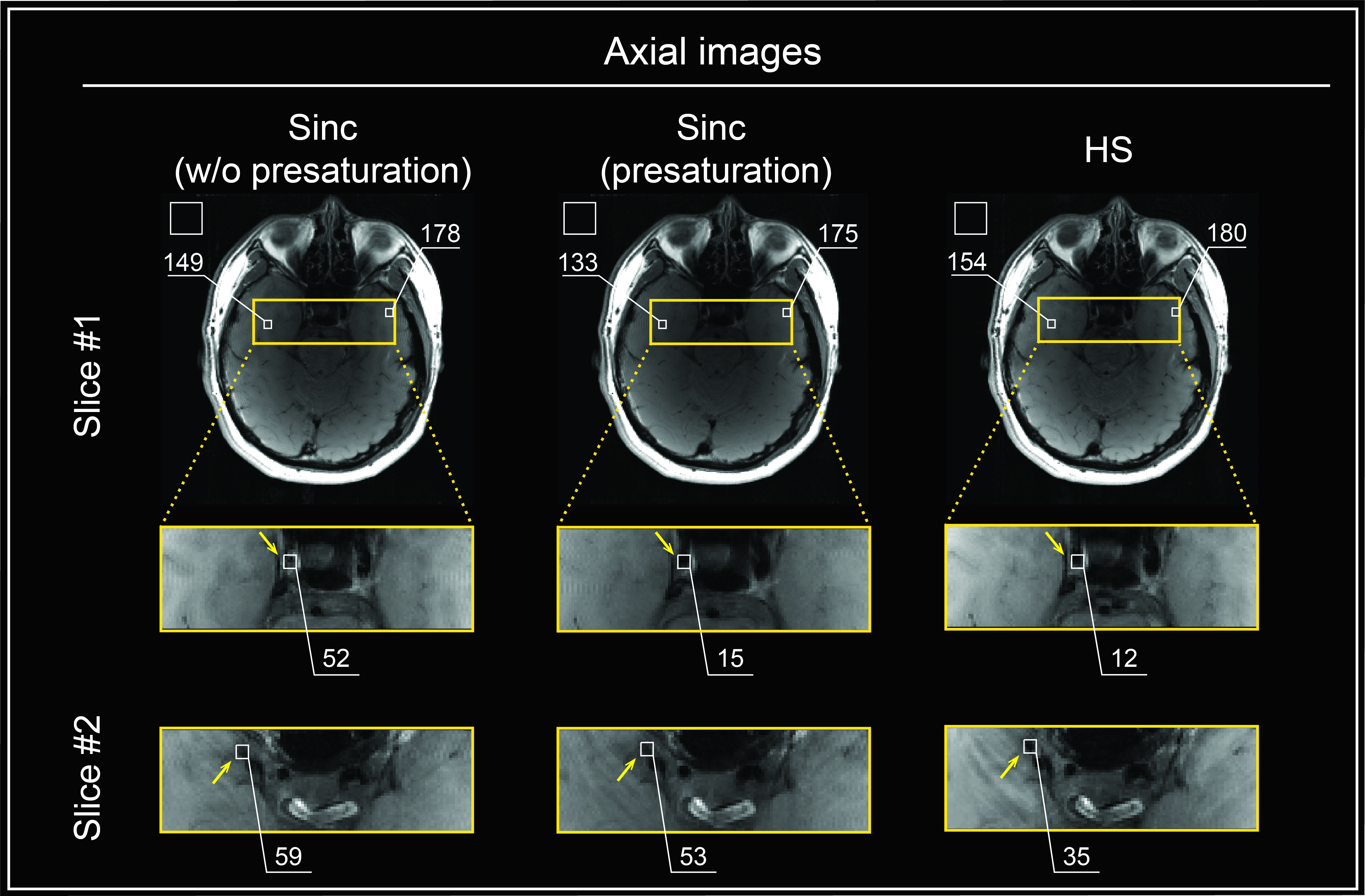
**

**Figure S4.** 2D spin-echo human brain images acquired using a head coil at 3T. Axial images obtained using 5-lobe sinc pulses without/with presaturation and HS pulses without presaturation. Arterial blood signals in the cavernous internal carotid arteries were effectively suppressed in the image acquired with HS pulses (yellow arrows).

**Supporting Information IV. Comparison of flow suppression performance of HS pulses and sinc pulses with presaturation**

Although presaturation has not been frequently used for liver DWI using spin-echo EPI sequence since blood signals are inherently suppressed due to the long TE, we compared the flow suppression performance of the proposed sequence and conventional sequence using sinc pulses with presaturation in liver DWI. For π/2 excitation and π refocusing, HS pulses with *β*_1_ = *β*_2_ = 5.3, *T*_p,1_ = *T*_p,2_ = 10.24 ms, and *BW*_1_/2π = 2*BW*_2_/2π = 7.66 kHz were used, respectively. The scan parameters were as follows: TR/TE = 1600 ms/73 ms, FOV = 280 × 280 mm^2^, matrix size = 128 × 128, slice thickness = 7.5 mm, *b*(average) = [100(2), 400(4)] s/mm^2^, readout BW = 1502 Hz/px, acceleration factor = 2 (GRAPPA), 6/8 partial Fourier acquisition, and diffusion encoding direction = orthogonal. Fat suppression was performed using spectral attenuated inversion recovery (SPAIR). Prospective respiratory gating was used.

As shown in Figure S5, liver images with *b* = 100 s/mm^2^ acquired using sinc pulses showed bright signals from moving blood flow even with presaturation, whereas those acquired with HS pulses showed effective suppression of these signals (yellow arrows).

**
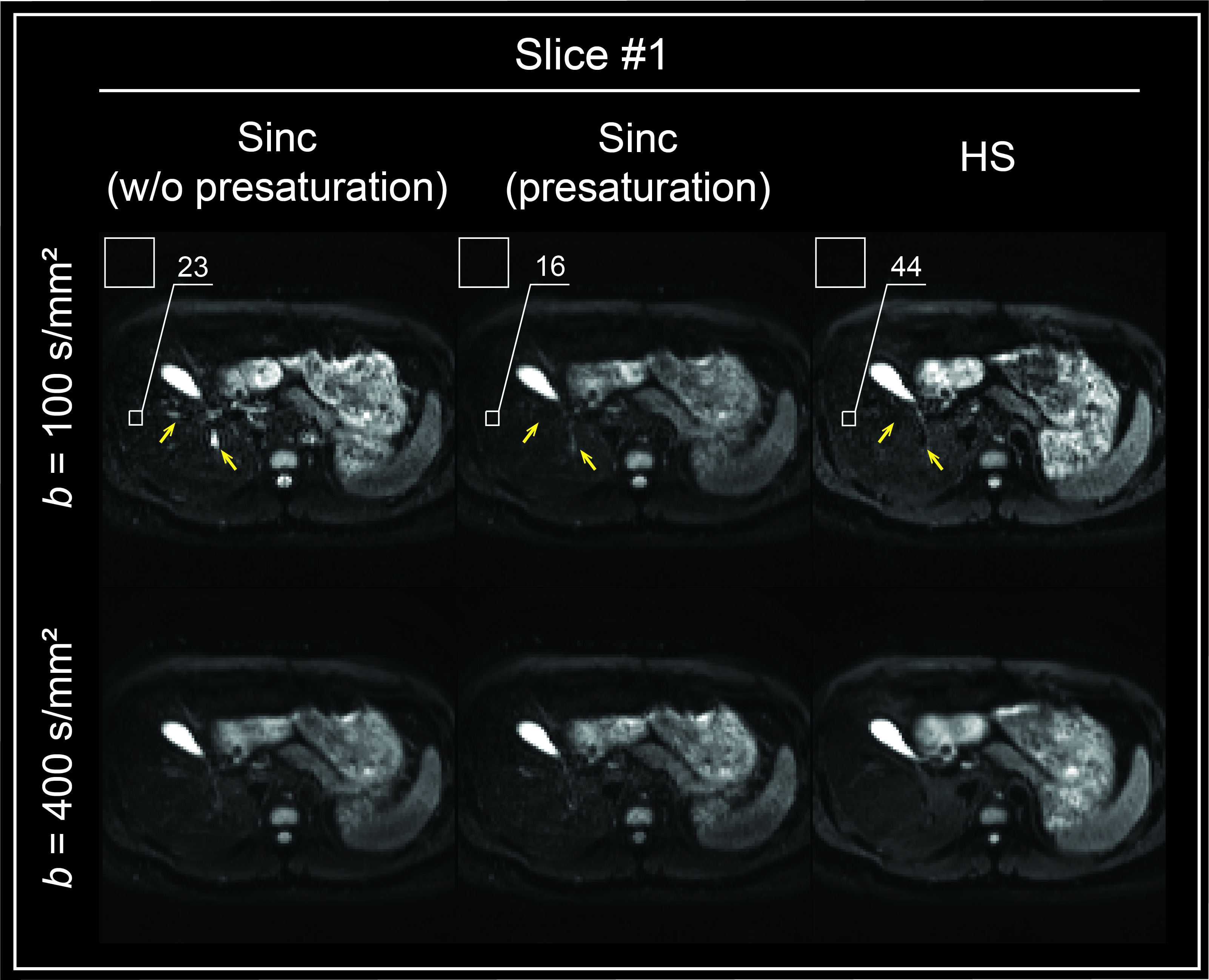
**

**Figure S5.** 2D spin-echo diffusion EPI human liver images acquired using a body coil at 3T. A bipolar motion-compensated diffusion-weighted gradient was used for diffusion encoding. Axial images obtained using 5-lobe sinc pulses without/with presaturation and HS pulses without presaturation. Owing to the adiabatic π refocusing HS pulse, the image acquired with HS pulses shows high SNR in the peripheral area of the liver, with better definition of abdominal structures at the periphery. In the image acquired with sinc pulses, bright blood signals were prominent, particularly in the abdominal aorta and veins. In contrast, these signals were effectively suppressed in the image acquired with HS pulses (yellow arrows).

**Supporting Information V. Limitations of being susceptible to motion**

In human liver DWI, we used higher bandwidth (*BW*_1_/2π = 2*BW*_2_/2π = 6.13 kHz, *v*_null_ ≈ 3.0 cm/s) for excitation and refocusing compared to human brain imaging (*BW*_1_/2π = 2*BW*_2_/2π = 4.14 kHz), in order to suppress the relatively slow blood flow in the liver compared to the brain (e.g., hepatic venous flow, mean hepatic vein velocity ≈ 5.1 ± 2.1 cm/s, Sudhamshu KC, Matsutani S, Maruyama H, Akiike T, Saisho H. Doppler study of hepatic vein in cirrhotic patients: Correlation with liver dysfunction and hepatic hemodynamics. *WJG*. 2006; 12(36): 5853-5858). In this case, the signal intensity as a function of flow velocity at TE can be represented as Figure S6 in the same manner as Figure 3A. However, the velocity of respiratory and cardiac motion has been reported to be approximately 1.5 cm/s to 3.5 cm/s (Boussuges A, Chaumet G, Boussuges M, Menard A, Delliaux S, Brégeon F. Ultrasound assessment of the respiratory system using diaphragm motion-volume indices. Front. Med. 2023. 10:1190891. doi: 10.3389/fmed.2023.1190891 and Mundigler G, Zehetgruber M. Tissue Doppler Imaging: Myocardial Velocities and Strain – Are there Clinical Applications?. J. Clin. Basic. Cardiol. 2002; 5: 125-132), which are within the range in which signals are considerably suppressed under the above condition. Therefore, although the proposed technique can effectively suppress the bright blood signals in liver DWI, the image quality may be degraded in terms of SNR and could be exacerbated in the uppermost part of the liver, close to the apex of the heart and lungs, as shown in Figure 7B. In this regard, pulse parameters such as bandwidth and pulse duration should be carefully optimized to balance blood flow suppression and motion-susceptibility.

Furthermore, we compared the SNR in the right lobe of the liver, where signal intensity appeared to be lower when using HS pulses than when using sinc pulses, for 11 consecutive slices (Figure S7A, slice #10 ~ #20). As shown in Figure S7B, the SNR was higher when using HS pulses compared to sinc pulses from slice #10 to slice #17. However, starting from slice #18 which corresponds to the uppermost part of the liver, the trend reversed and sinc pulses produced higher SNR than HS pulses in slices #18 to #20. In this regard, the proposed technique is highly recommended for use except in the uppermost part of the liver to prevent signal loss.


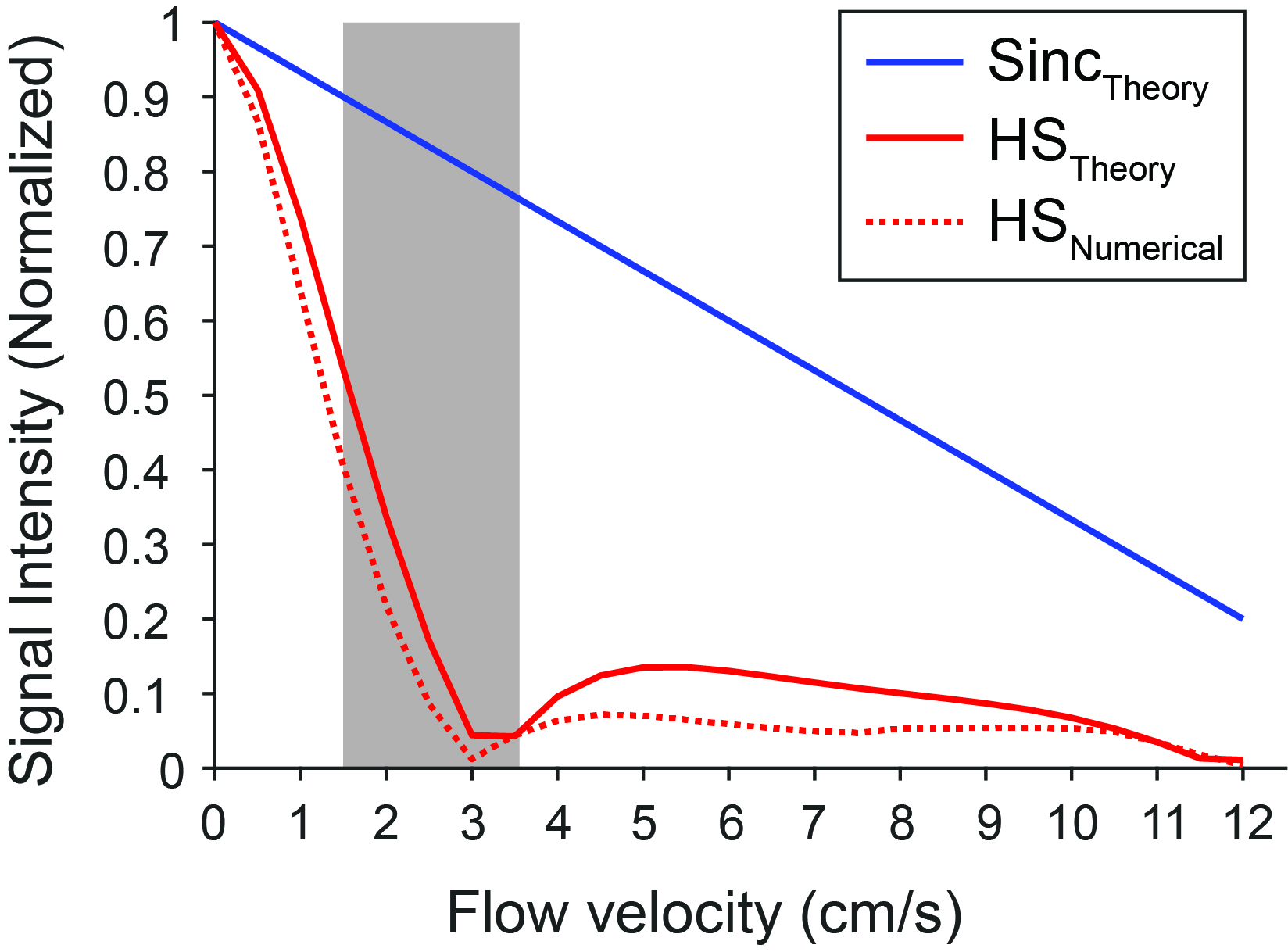


**Figure S6.** Signal intensity as a function of flow velocity at TE when sinc and HS pulses are used for both π/2 excitation and π refocusing. HS pulses with *β*_1_ = *β*_2_ = 5.3, *T*_p,1_ = *T*_p,2_ = 5.12 ms, *BW*_1_/2π = 2*BW*_2_/2π = 6.13 kHz were used for π/2 excitation and π refocusing HS pulses, respectively. Scan parameters were as follows: TE = 80 ms and slice thickness = 6 mm.


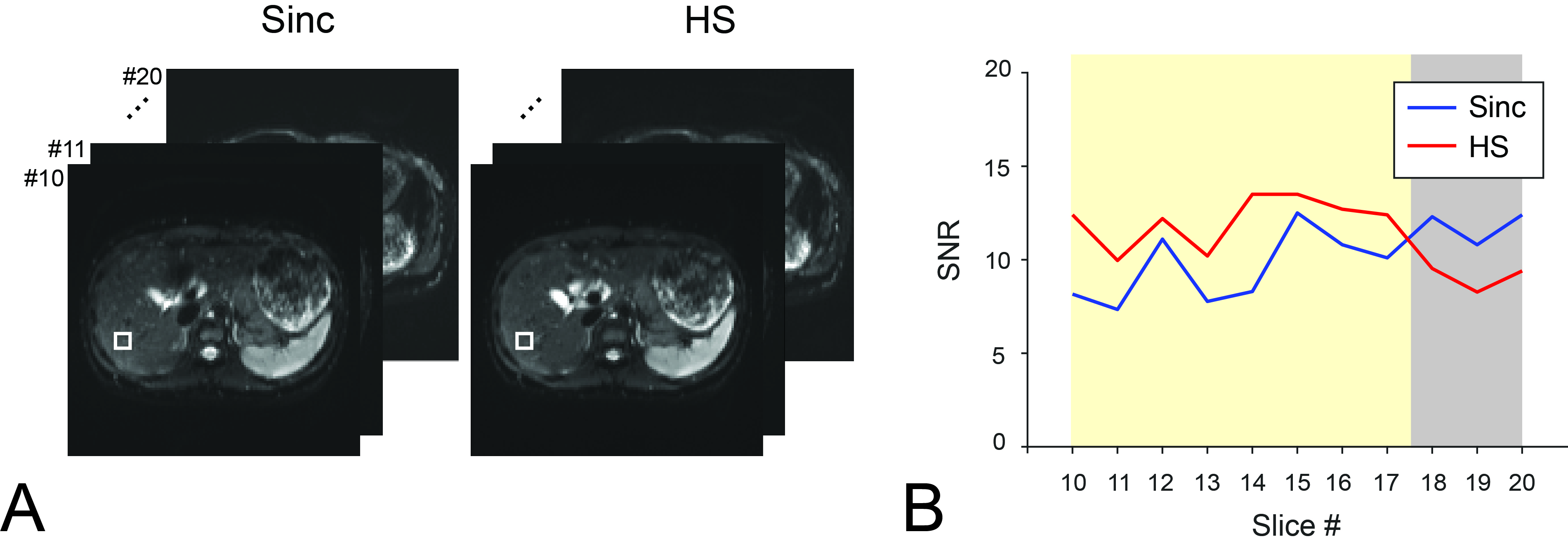


**Figure S7.** SNR comparison of liver images acquired by 2D spin-echo DWI using sinc pulses and HS pulses. (A) Selected square region of interests (ROIs) in the posterior part of the right lobe of the liver. (B) SNR in each slice when using sinc pulses and HS pulses. SNR was simply calculated as the average signal intensity divided by the standard deviation within the ROI.
